# Efficient CO_2_-Reducing Activity of NAD-Dependent Formate Dehydrogenase from *Thiobacillus* sp. KNK65MA for Formate Production from CO_2_ Gas

**DOI:** 10.1371/journal.pone.0103111

**Published:** 2014-07-25

**Authors:** Hyunjun Choe, Jeong Chan Joo, Dae Haeng Cho, Min Hoo Kim, Sang Hyun Lee, Kwang Deog Jung, Yong Hwan Kim

**Affiliations:** 1 Department of Chemical Engineering, Kwangwoon University, Seoul, Republic of Korea; 2 Department of Microbial Engineering, Konkuk University, Seoul, Republic of Korea; 3 Clean Energy Research Center, Korea Institute of Science and Technology, Seoul, Republic of Korea; University of Alberta, Canada

## Abstract

NAD-dependent formate dehydrogenase (FDH) from *Candida boidinii* (CbFDH) has been widely used in various CO_2_-reduction systems but its practical applications are often impeded due to low CO_2_-reducing activity. In this study, we demonstrated superior CO_2_-reducing properties of FDH from *Thiobacillus* sp. KNK65MA (TsFDH) for production of formate from CO_2_ gas. To discover more efficient CO_2_-reducing FDHs than a reference enzyme, i.e. CbFDH, five FDHs were selected with biochemical properties and then, their CO_2_-reducing activities were evaluated. All FDHs including CbFDH showed better CO_2_-reducing activities at acidic pHs than at neutral pHs and four FDHs were more active than CbFDH in the CO_2_ reduction reaction. In particular, the FDH from *Thiobacillus* sp. KNK65MA (TsFDH) exhibited the highest CO_2_-reducing activity and had a dramatic preference for the reduction reaction, i.e., a 84.2-fold higher ratio of CO_2_ reduction to formate oxidation in catalytic efficiency (*k*_cat_/*K*_B_) compared to CbFDH. Formate was produced from CO_2_ gas using TsFDH and CbFDH, and TsFDH showed a 5.8-fold higher formate production rate than CbFDH. A sequence and structural comparison showed that FDHs with relatively high CO_2_-reducing activities had elongated N- and C-terminal loops. The experimental results demonstrate that TsFDH can be an alternative to CbFDH as a biocatalyst in CO_2_ reduction systems.

## Introduction

Reducing the atmospheric CO_2_ level has received a great deal of attention recently as an approach to combat global warming and fossil-fuel shortages, but this process remains challenging. Biological CO_2_ fixation is one of the most important approaches to solving these problems. Enzymatic CO_2_ reduction has been examined extensively as a promising approach to greenhouse gas fixation and the production of renewable fuels and chemicals [1]–[3]. The enzymatic reduction of CO_2_ using FDHs has been widely studied for the production of valuable chemicals, such as formic acid and methanol [4], [5]. Formic acid is considered to be a promising replacement for methanol in miniature fuel cells [6]. Formic acid has been produced by the hydrolysis of methyl formate, which is synthesized via methanol carbonylation in commercial processes. Therefore, it would be environmentally attractive to prepare formic acid from CO_2_ gas by enzymatic biotransformation.

Many efforts have been made to develop CO_2_-reducing chemical catalysts, and recent research on chemical catalysts has led to improved rates for CO_2_ reduction [7], [8]. However, chemical catalysts require harsh reaction conditions and/or expensive metals, such as ruthenium, rhodium, and iridium [9]–[11]. In contrast to a chemical CO_2_ reduction, CO_2_ can be reduced by enzymes under mild conditions. There are few biocatalysts capable of biological CO_2_ fixation, e.g. pyruvate decarboxylase (EC 4.1.1.1), carbonic anhydrase (EC 4.2.1.1), and FDH (EC 1.2.1.2). Pyruvate decarboxylase can catalyze the reversible conversion of pyruvate into CO_2_ and acetaldehyde and thus requires equimolar acetaldehyde for the conversion of CO_2_ into pyruvate [3]. It should be noted that carbonic anhydrase can catalyze the rapid interconversion of CO_2_ and bicarbonate but this is not a real CO_2_ reduction reaction but a CO_2_ hydration reaction [12], [13]. However, FDH can reduce CO_2_ to formate without any other organic chemicals, and formate can be sequentially reduced to formaldehyde and methanol by coupling aldehyde dehydrogenase and alcohol dehydrogenase reactions [4]. Therefore, FDH has been widely adopted in CO_2_ reduction reactions [4], [14]–[16]. FDH can be divided into two groups, NAD-independent or NAD-dependent. NAD-independent FDHs have a high CO_2_-reducing activity but include extremely oxygen-labile catalytic components, such as metal ions (tungsten or molybdenum), iron-sulfur clusters, and selenocysteine, making these FDHs unsuitable for industrial applications [17]–[20]. Recently, NAD-dependent FDHs have been utilized in CO_2_ reduction systems as an alternative to NAD-independent FDHs. In particular, CbFDH is commercially available and has been widely adopted as a CO_2_-reducing biocatalyst in electrochemical, photochemical, and enzymatic reactions [4], [14], [16] as well as a NADH-regenerating biocatalyst in enzyme-coupled reaction systems [21]–[24]. However, the CO_2_-reducing activity of CbFDH is still very low for practical applications, and thus it is necessary to discover more efficient FDHs than CbFDH.

In this study, we report superior CO_2_-reducing performance of TsFDH. We selected five FDHs based on their biochemical properties, e.g. acidic optimum pH, specific activity, and stability, and investigated their feasibility as CO_2_-reducing biocatalysts. Enzyme activities in formate oxidation and CO_2_ reduction were measured, and the ternary complex model was applied to understand the characteristics of FDHs. Finally, the concentration of formate produced form CO_2_ gas using TsFDH and CbFDH was compared. Based on these experimental results, TsFDH can be a good substitute for CbFDH as an efficient CO_2_-reducing biocatalyst.

## Materials and Methods

### Materials

The *fdh* genes used in this study were synthesized with an additional C-terminal hexa-histidine sequence by GenScript (USA). Restriction enzymes were purchased from Takara (Japan). *Pfu* DNA polymerase and ligase were purchased from New England Biolabs (USA). The pET-23b(+) vector and competent cell *E. coli* DH5α/BL21 (λDE3) were purchased from Novagen (USA). Ni-NTA resin was purchased from Qiagen (USA). SYPRO Orange dye was purchased from Invitrogen (USA). CbFDH and all chemicals used in enzyme reactions were purchased from Sigma-Aldrich (USA).

#### Cloning, expression, and purification of FDHs

The *fdh* genes containing a C-terminal hexa-histidine sequence in the pUC57 vector were PCR-amplified using primers (Table S1). The amplified fragments were digested by *Nhe* I and *EcoR* I and then cloned into a pET-23b(+) vector. The sequence of recombinant plasmids was confirmed by DNA sequencing (Cosmogenetech, Korea). The recombinant plasmids were chemically transformed into competent *E. coli* BL21 (λDE3). Expression and purification of the FDHs were performed as previously described [25]. Ni-NTA resin was used to purify the FDHs with a C-terminal hexa-histidine tag. To obtain pure enzymes, the recombinant FDHs were washed with 50 ml of wash buffer (50 mM NaH_2_PO_4_ pH 7.0, 300 mM NaCl, 40 mM imidazole), and the purity of the FDHs was determined by 12% SDS-PAGE.

#### Determination of conformational stability

The conformational stability of the FDHs was determined by differential scanning fluorimetry (DSF) using a real-time quantitative PCR thermal cycler (LightCycler 480, Roche, USA). The DSF method can measure the temperatures at the midpoint of a protein's melting transition (T_m_) by monitoring their thermal unfolding. SYPRO Orange (20x) and 10 to 30 µM of purified FDHs were used in 96-well qPCR microplates. The T_m_ values were determined by heating from 20 to 95°C at a 1°C/min scan rate as previously described [26].

#### Activity assay of FDHs

The FDH activity was determined by monitoring the absorbance changes at 340 nm during the redox reactions catalyzed by the FDHs at 25°C and different pH values (5.5∼7.0). One unit of oxidation activity was defined as the amount of enzyme required to produce 1 µmol of NADH per minute under standard conditions. The oxidation of formate was conducted using an assay solution (2 ml of a 100 mM sodium phosphate buffer) containing 20 µg of FDH, 200 mM sodium formate, and 2 mM NAD^+^. One unit of reduction activity was defined as the amount of enzyme required to consume 1 µmol of NADH per minute under standard conditions. The reduction of CO_2_ was conducted using an assay solution (2 ml of a 100 mM sodium phosphate buffer) containing 3.0 mg of FDH, 50 mM NaHCO_3_, and 0.15 mM NADH. In the case of the CO_2_ reduction reaction, NaHCO_3_ was used as a substrate to supply CO_2_ because the concentration of gaseous CO_2_ cannot be accurately determined. Therefore, the substrate concentration for CO_2_ reduction was represented as that of NaHCO_3_. A relatively large amount of enzyme was used for CO_2_ reduction due to the very low CO_2_-reducing activity of CsFDH. All activities were calculated by subtracting the decomposition rate of NADH in the absence of the enzyme.

#### Determination of kinetic parameters

The saturation concentration of NADH required for CO_2_ reduction was too high to adopt the steady-state model and thus the ternary complex model [27] was applied with low concentrations of substrate and cofactors. The kinetic parameters were determined according to the sequential mechanism model proposed by Cleland [28]. This mechanism was described as follows (1): 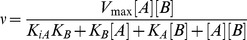
(1)where *V*_max_ is the maximum reaction rate, *K*_A_ and *K*_B_ are the Michaelis constants for NADH (reduction)/NAD^+^ (oxidation) and NaHCO_3_ (reduction)/formate (oxidation), respectively, in the presence of saturating concentrations of the other substrate. *K*_iA_ is the dissociation constant for the enzyme-cofactor complex. The model was fitted to the experimental data using SigmaPlot (Version 10.0, Systat Software Inc., USA).

The kinetic parameters of the FDH-catalyzed oxidation and reduction reactions were determined in 100 mM sodium phosphate buffer (pH 7.0) with a 1.1 ml reaction volume at 25°C. To perform the formate oxidation reaction, the content of FDHs was fixed at 5 µg, while the concentration of NAD^+^ varied from 0.1 to 0.3 mM and that of sodium formate varied from 10 to 50 mM. The reduction reaction of CO_2_ was achieved with 10 and 100 µg of TsFDH and CbFDH, respectively, and the ranges of concentrations for NADH and sodium bicarbonate were same as those of NAD^+^ and sodium formate for the formate oxidation reaction. The initial rates of the reactions were determined by measuring the change in NADH.

#### Formate production from CO2 gas

Sodium phosphate buffer (100 mM, pH 7.0) was purged with CO_2_ for 1 h and adjusted to pH 7.0 with NaOH. Formate production reactions were performed by 4.4 µM FDHs (1.0 mg of TsFDH or 0.9 mg of CbFDH) with 10 mM NADH. The total volume of reaction solution was 5 ml. The reaction solution was mixed with a magnetic stir bar at 200 rpm and was purged continuously with CO_2_ at a flow rate of 50 ml/min during the CO_2_ reduction reactions. The amount of formate produced was determined by high-pressure liquid chromatography (HPLC) equipped with an Aminex HPX 87-H ion exclusion column (300×7.8 mm) and a refractive index detector (RID). The temperature of the column and the RID detector was set to 35°C. H_2_SO_4_ solution (5 mM) was used as the mobile phase at a flow rate of 0.6 ml/min. Forty microliters of the sample was injected into HPLC for analysis.

## Results

### Production of recombinant NAD-dependent FDHs

**Figure 1:**
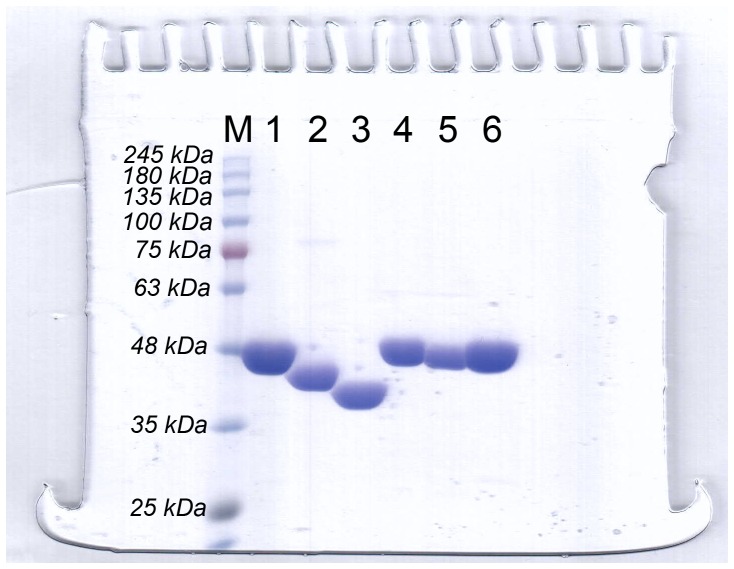
SDS-PAGE analysis of purified FDHs. Lane M: molecular mass marker, 1: AaFDH, 2: CbFDH, 3: CsFDH, 4: MsFDH, 5: PsFDH, 6: TsFDH.

The *fdh* genes encoding FDHs from *Ancylobacter aquaticus* KNK607M (AaFDH, BAC65346.1) [29], *Ceriporiopsis subvermispora* (CsFDH, BAF98206.1) [30], *Moraxella* sp. C-1 (MsFDH, CAA73696.1), *Paracoccus* sp. 12-A (PsFDH, BAB64941.1) [31], and *Thiobacillus* sp. KNK 65MA (TsFDH, BAC92737.1) [32] as well as the gene for CbFDH (CAA09466.2) were synthesized, and all recombinant FDHs were successfully expressed with a C-terminal hexa-histidine tag in *E. coli*. The SDS-PAGE gel exhibited the molecular masses of the single subunits that were identical to the calculated molecular mass of AaFDH (45 kDa), CbFDH (41 kDa), CsFDH (40 kDa), MsFDH (45 kDa), PsFDH (45 kDa), and TsFDH (45 kDa) and showed that the recombinant FDHs had a high purity (Fig. 1).

The conformational stability of the FDHs was determined by evaluating their thermal unfolding using the DSF method as previously described [25], and all FDHs were sufficiently stable (T_m_ = 42.3∼61.8°C) under ambient conditions (Fig. S1).

### Enzyme activities for formate oxidation and CO_2_ reduction

**Figure 2:**
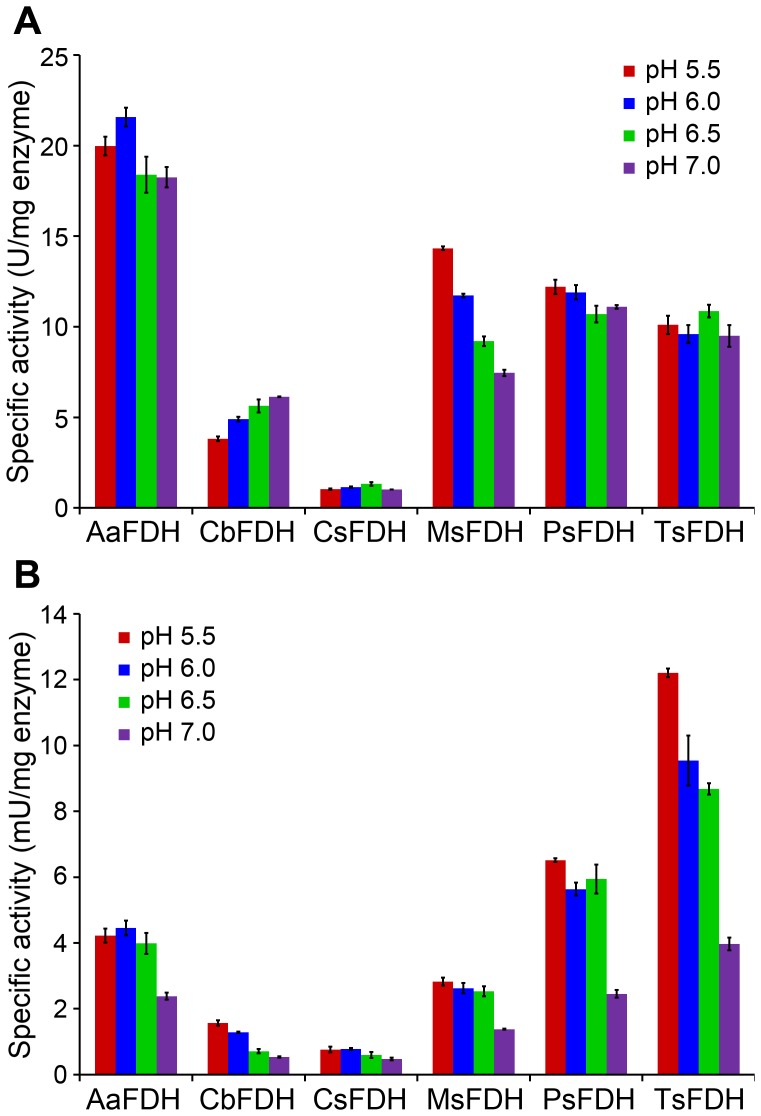
Enzyme activities of FDHs. Enzyme activities of FDHs for A) formate oxidation and B) CO_2_ reduction at different pH values.

The activities of the recombinant FDHs, including CbFDH, in the oxidation and reduction reactions are shown in Fig. 2. All enzyme activities were determined by measuring the initial rates of reaction. All FDHs except for CsFDH had higher enzyme activities in the formate oxidation reaction than CbFDH. CsFDH activity was 1.0∼1.3 U/mg enzyme at all tested pH ranges (Fig. 2A). AaFDH exhibited the highest activity, and its enzyme activity was 5.2-fold (20.0 U/mg enzyme) and 3.0-fold (18.3 U/mg enzyme) higher than those of CbFDH at pH 5.5 and 7.0, respectively. The FDHs did not exhibit a drastic change in formate oxidation activity at the tested pH ranges. The CO_2_-reducing activities of FDHs are shown in Fig. 2B. Compared to the formate oxidation activity, all FDHs exhibited low enzyme activities (0.5∼12.2 mU/mg) in the CO_2_ reduction reaction and had an acidic optimum pH (pH 5.5 or 6.0). Four screened FDHs (AaFDH, MsFDH, PsFDH, and TsFDH) were more active over the pH range of 5.5 to 7.0 than CbFDH, and TsFDH had the highest CO_2_-reducing activity. Its activities were at least 7.3-fold higher (12.2, 9.5, 8.7, and 4.0 mU/mg enzyme) than those of CbFDH (1.6, 1.3, 0.7, and 0.5 mU/mg enzyme) at pH 5.5, 6.0, 6.5, and 7.0, respectively.

### Enzyme kinetics

**Figure 3:**
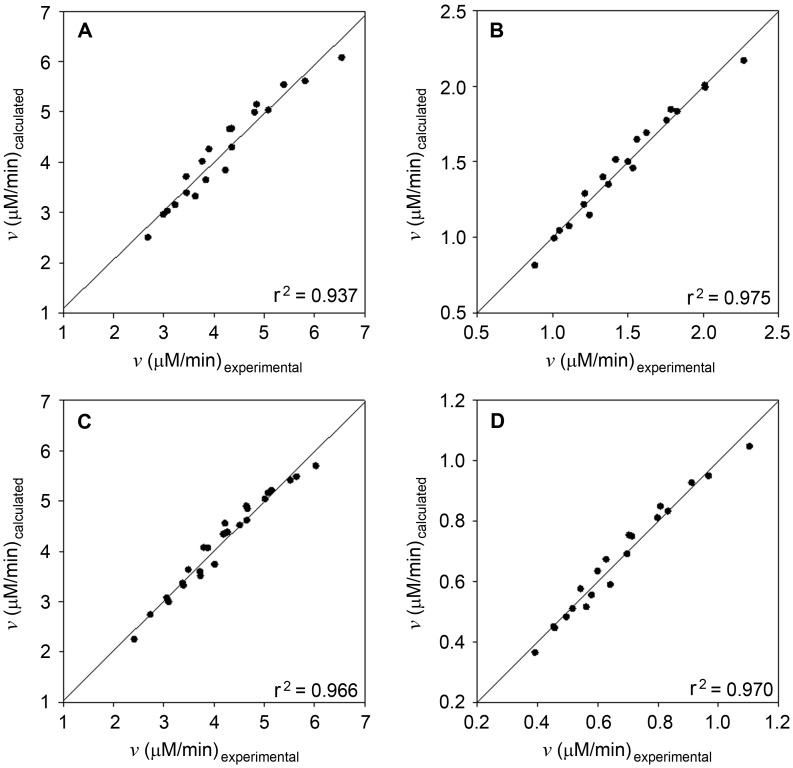
Determination of kinetic parameters. Correlation between the measured and calculated initial rates of TsFDH-catalyzed A) formate oxidation and B) CO_2_ reduction; CbFDH-catalyzed C) formate oxidation and D) CO_2_ reduction.

The kinetic parameters of TsFDH and CbFDH were determined at pH 7.0 because NADH is slowly degraded at acidic pHs [33], resulting in unreliable kinetic values at acidic pHs. Initial rates were measured with respect to cofactors at various fixed concentrations of substrates. Kinetic parameters for the forward and reverse reaction were obtained by fitting the initial rates to equation (1) with none-linear regression (Table 1). Regression coefficients (r^2^) for all reactions were over 0.937 (Fig. 3). In the formate oxidation reaction, TsFDH showed a lower substrate binding affinity (*K*_B_, 16.24 mM) and a higher turnover number (*k*_cat_, 1.769/s) than CbFDH (8.55 mM and 1.081/s, respectively), and both enzymes exhibited similar catalytic efficiencies (*k*_cat_/*K*_B_). In the CO_2_ reduction reaction, however, TsFDH had a 3.4-fold higher substrate-binding affinity (*K*_B_, 9.23 mM), a 21.2-fold higher turnover number (*k*_cat_, 0.318/s), and a 85-fold higher catalytic efficiency (*k*_cat_/*K*_B_, 0.034/mM·s) than CbFDH (31.28 mM, 0.015/s, and 0.0004/mM·s, respectively). TsFDH exhibited a dramatic preference for the reduction reaction with a 84.2-fold higher catalytic efficiency ratio of CO_2_ reduction to formate oxidation than that of CbFDH. The recombinant CbFDH expressed in *E. coli* and commercial CbsFDH (Sigma-Aldrich) showed similar catalytic efficiencies for the forward and reverse reaction (data not shown) and thus only the data from the recombinant CbFDH was reported in this paper.

**Table 1:** The kinetic parameters of FDHs[a].

	*K*_A_ (mM)	*K*_iA_ (mM)	*K*_B_ (mM)	*k*_cat_ (1/s)	*k*_cat_/*K*_B_ (1/mM·s)	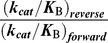
TsFDH[b]	0.281±0.081	0.005±0.002	16.24±5.39	1.769±0.441	0.109	3.2×10^−1^
TsFDH[c]	0.264±0.076	0.126±0.053	9.23±3.98	0.318±0.051	0.034	
CbFDH[b]	0.102±0.030	0.134±0.087	8.55±2.56	1.081±0.109	0.126	3.8×10^−3^
CbFDH[c]	0.512±0.186	0.014±0.010	31.28±8.05	0.015±0.005	0.0004	

[a] Kinetic parameters for the forward (formate oxidation) and reverse (CO_2_ reduction) reactions were calculated by fitting the initial rates to equation (1).

[b] Formate oxidation (A: NAD^+^, B: sodium formate).

[c] CO_2_ reduction (A: NADH, B: sodium bicarbonate).

### Formate production through enzymatic CO_2_ reduction

**Figure 4:**
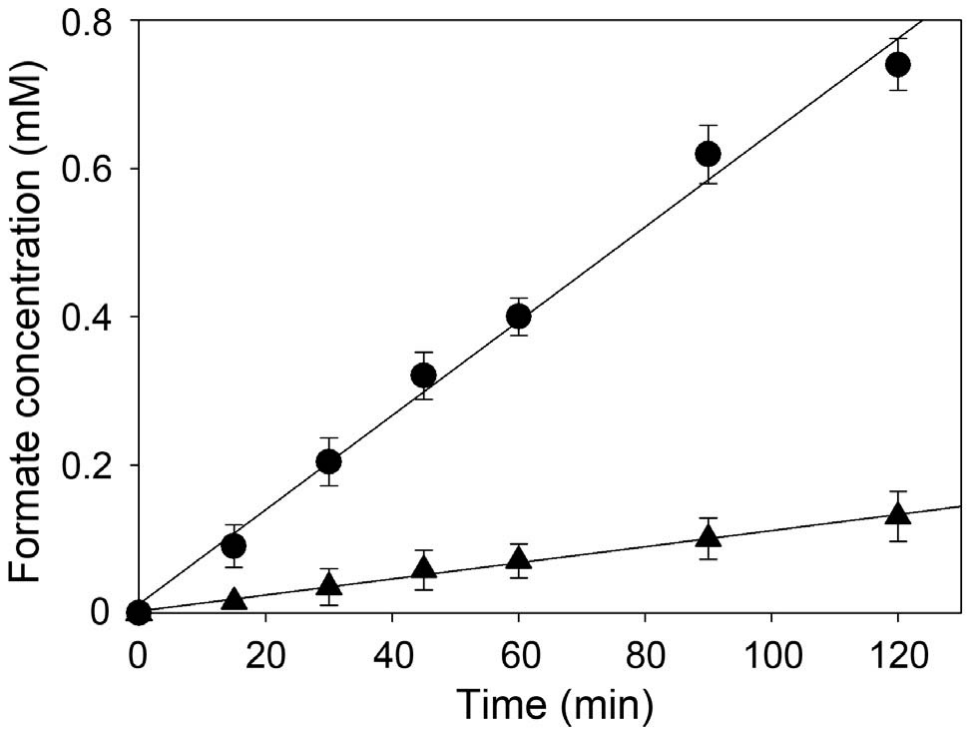
Formate production through FDH-catalyzed CO_2_ reduction. Formate production by (•) TsFDH and (▴) CbFDH in 100 mM sodium phosphate buffer, pH 7.0.

Formate was produced from CO_2_ gas using TsFDH and CbFDH. The amount of formate produced through the enzymatic CO_2_ reduction is shown in Fig. 4. TsFDH and CbFDH produced 0.74 mM and 0.13 mM formate in 120 min, respectively. The formate production rate of TsFDH and CbFDH was linear, with a constant rate of 0.023/s and 0.004/s, respectively. TsFDH showed a 5.8-fold higher formate production rate than CbFDH, which is consistent with the results shown in Fig. 2B.

## Discussion

FDHs can catalyze the conversion of CO_2_ and formate and thus are of great interest as CO_2_-reducing biocatalysts for CO_2_ sequestration and for the production of formate as a source of fuels and commodity chemicals. NAD-independent FDHs can drive the CO_2_ reduction reaction with electrons supplied from an electrode and artificial electron mediators, such as methyl viologen, exhibiting very high CO_2_-reducing catalytic efficiency [20], [34]. Despite this advantage, the use of NAD-independent FDHs in CO_2_ reduction systems does not appear to be practical due to the requirement for complicated catalytic components, such as molybdopterin cofactor, iron-sulfur clusters, and selenocysteine, in addition to their oxygen-labile activity, which results in insoluble and inactive expression in *E. coli* [35]–[37]. Recently, K. Schuchmann and V. Müller reported that a hydrogen-dependent carbon dioxide reductase (HDCR) from *Acetobacterium woodii* can catalyze reduction of CO_2_ to formate with very high activity [38]. However, it is also very unstable under aerobic conditions as it has the catalytic components (Table S2). In contrast to NAD-independent FDHs and HDCR, NAD-dependent FDHs are oxygen-stable and can be highly expressed in *E. coli* as demonstrated in this study, but their practical applications in CO_2_-reduction systems are still limited due to their low CO_2_-reducing activities.

In this study, we attempted to identify FDHs that are superior to a conventional CO_2_-reducing biocatalyst, i.e., CbFDH. FDHs suitable for CO_2_ reduction were screened from BRENDA (BRaunschweig ENzyme Database, http://www.brenda-enzymes.org) [39] based on their optimum pH. The catalytic mechanism of formate oxidation by NAD-dependent FDHs has been demonstrated to involve direct hydride transfer from formate to the C4 atom of the nicotine amide ring of NAD^+^ [40], [41]. However, it remains unclear whether NAD-dependent FDHs use a proton-relay system in the CO_2_ reduction reaction. The abundance of protons would be favorable for the reduction of many chemicals [42], [43]. Moreover, Peacock and Boulter reported that FDH from *Phaseolus aureus* (PaFDH) had 19.7-fold higher CO_2_-reducing activity at pH 6.3 than at pH 8.0 (710 pmol/min vs. 36 pmol/min) with approximately equivalent concentrations of enzyme and substrate and showed a 19.6-fold lower ratio of the rates of the forward (formate oxidation) and reverse (CO_2_ reduction) reaction (2,300 at pH 6.3 vs. 45,000 at pH 8.0) [27]. These results imply that FDHs with an acidic optimum pH would be more efficient for CO_2_ reduction than FDHs with neutral or alkaline optimum pH. In the present study, the preliminary experiment also revealed that CbFDH has a higher CO_2_-reducing activity at an acidic pH (1.6 mU/mg enzyme at pH 5.5) than at a neural pH (0.5 mU/mg enzyme at pH 7.0), whereas it exhibited higher formate-oxidation activity at a neutral pH (6.1 U/mg enzyme at pH 7.0) than at an acidic pH (3.8 U/mg enzyme at pH 5.5) (Fig. 2). In addition, it has been reported that CO_2_, bicarbonate (HCO_3_^−^), and carbonate (CO_3_^2−^) are dominant at acidic, neutral, and alkaline pHs, respectively [44]. As mentioned above, CO_2_ reduction at an acidic pH has apparent advantages over reduction under neutral or alkaline pH conditions, including the presence of abundant protons, a high CO_2_ fraction [42], and higher CO_2_-reducing activity of FDHs. Therefore, we selected five NAD-dependent FDHs (AaFDH, CsFDH, MsFDH, PsFDH, and TsFDH) with an acidic optimum pH and comparable activity and stability to those of the reference enzyme CbFDH. All FDHs were successfully expressed and purified with Ni-NTA resin (Fig. 1). The five FDHs characterized in this study showed an acidic optimum pH for formate oxidation and CO_2_ reduction and moderate stability under ambient conditions (Table S3).

As shown in Fig. 2, AaFDH had the highest enzyme activity in the oxidation reaction, which was at least 3-fold higher than that of CbFDH. High formate oxidation activity and the DSF results (Table S3) indicate that AaFDH could be used as an alternative NADH-regenerating enzyme to CbFDH over a broad pH range. In the reduction reaction, all enzymes, including CbFDH, exhibited an acidic pH optimum, indicating that an acidic pH is more favorable than neutral pH for CO_2_ reduction. The lowest values for the ratio of the rates of the oxidation and reduction reaction were calculated as 4,600 at pH 6.5, 2,400 at pH 5.5, 1,400 at pH 5.5, 3,600 at pH 6.5, 1,800 at pH 6.5, and 820 at pH 5.5 for AaFDH, CbFDH, CsFDH, MsFDH, PsFDH, and TsFDH (obtained from Fig. 2), which are comparable to the value of PaFDH [27]. All FDHs showed better reduction activities at acidic pH than at neutral pH. TsFDH was the best biocatalyst in terms of CO_2_-reducing activity and the ratio of the rates of the oxidation and reduction reactions.

Previous enzyme kinetics revealed that FDHs have a sequential mechanism in which both substrates bind to the enzyme in a defined or random order before the products are released. Eukaryotic FDHs catalyze formate oxidation by an ordered kinetic mechanism, but bacterial FDHs follow a random mechanism with a rapid equilibrium [27], [40]. The Michaelis-Menten plots were obtained (Fig. S2). Saturation of NaHCO_3_ and NADH is very difficult for the CO_2_ reduction reaction. Saturation of NADH for enzyme-catalyzed CO_2_ reduction could not be achieved by high degradation rate of NADH and inhibitory effect of degraded compounds. The reaction rate was decreased with increasing NADH concentration of over 0.4 mM [45]. Low solubility of CO_2_ in buffer at atmospheric pressure also caused the difficulty of CO_2_ saturation for enzyme-catalyzed CO_2_ reduction. Therefore, typical Michaelis-Menten saturation plot which shows the convergence of velocity to *v*_max_ could not be obtained. However, kinetic constants could be obtained on the basis of generally acceptable rapid equilibrium assumption for enzyme-substrate complex. Double reciprocal plots of eukaryotic CbFDH and bacterial TsFDH were linear and gave intersecting patterns in the forward and reverse reaction (Fig. S3), indicating that the kinetic mechanism of both FDHs is sequential. Both FDHs exhibited a similar binding affinity for formate, which is comparable to that of typical NAD-dependent FDHs (1∼15 mM) [40]. Both FDHs had a similar catalytic efficiency (*k*_cat_/*K*_B_) in the oxidation of formate, but TsFDH showed a dramatic preference for CO_2_ reduction due to the 21.2-fold higher turnover number compared to CbFDH. These catalytic properties enable TsFDH to produce formate from CO_2_ gas more efficiently than CbFDH without the saturation of the reaction rate (Fig. 4). Conventional CO_2_ reduction systems using commercial CbFDH for the production of formate or methanol require *in situ* regeneration of NADH to drive CO_2_ reduction [4]. The formate production rate of TsFDH can be further improved by incorporating a NADH-regeneration system e.g., chemical, electrochemical, photochemical, or enzymatic method [46]. At an acidic pH, despite high TsFDH-catalyzed CO_2_ reduction reaction, it is evident that formate productivity will be gradually decreased due to degradation of NADH under acidic conditions [47]. Recently, various NAD analogs including thio-NAD, APAD, PAAD, and NAAD were found to be more efficient and stable than NAD in electrochemical regeneration systems [48]. Thus, these analogs need to be investigated as an alternative cofactor to overcome the instability NADH at an acidic pH.

**Figure 5:**
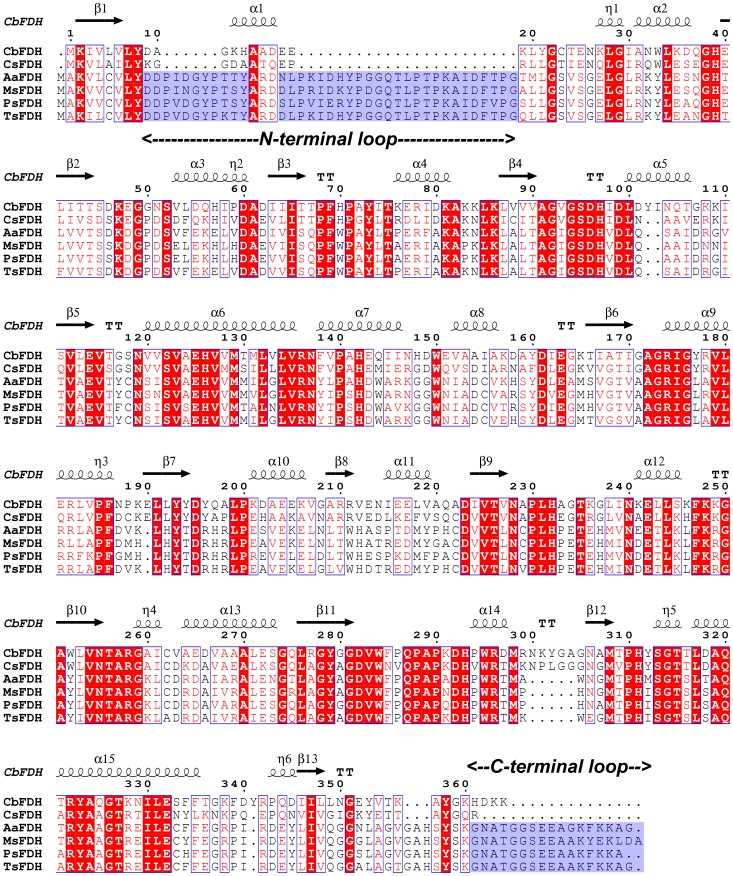
Sequence alignment of NAD-dependent FDHs. Amino acid sequences of FDH from yeast: CbFDH; FDH from fungi: CsFDH; FDHs from bacteria: AaFDH, MsFDH, PsFDH, and TsFDH. The blue background indicates the additional sequence regions for the N- and C-terminal loops of bacterial FDHs. Conservative amino acids are represented in red box and secondary structure elements are assigned according to the structure of CbFDH (pdb code: 2FSS).

**Figure 6:**
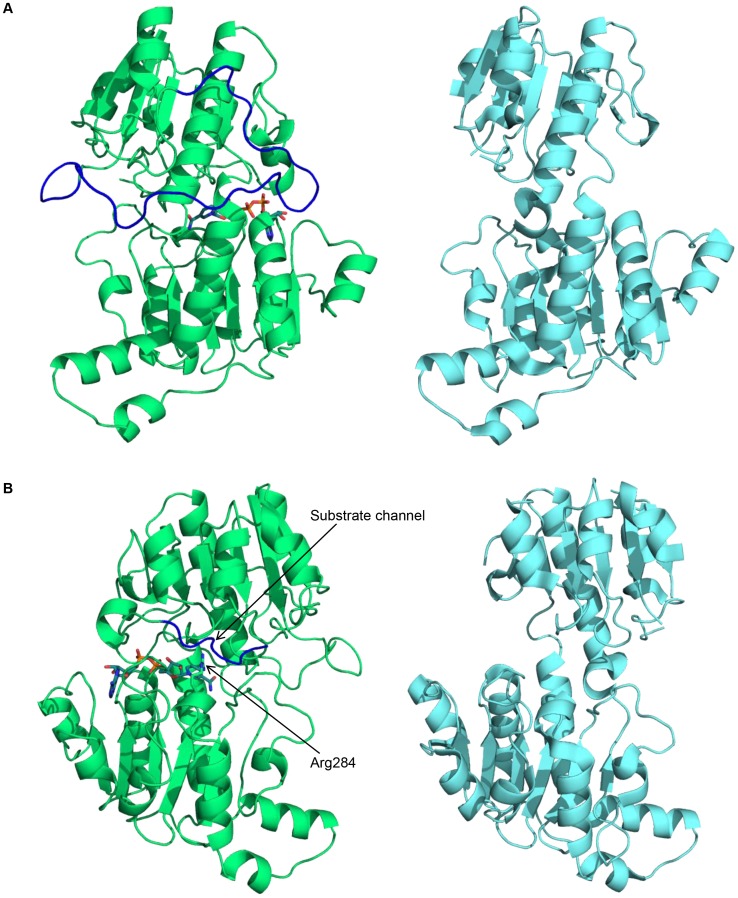
Structural comparison of TsFDH and CbFDH. Structural comparison of the A) N- and B) C-terminal loops of TsFDH (green, modeled using 2NAD) and CbFDH (cyan, pdb code: 2FSS). The elongated N- and C-terminal loops are shown in blue.

Tishkov and Popov performed structural and multiple sequence alignment of eukaryotic FDHs and bacterial FDHs and found that bacterial FDHs have an addition loop in the N-terminal end, which may have an important role in the discrimination of the kinetic mechanism of the two different groups of FDHs [40]. In this study, it should be noted that four bacterial FDHs showed higher CO_2_-reducing activities than two eukaryotic FDHs. Multiple sequence alignment of six FDHs was conducted using CLUSTAW2 and ESPript 2.2 [49], [50] (Fig. 5). The FDHs tested in this study can be classified into two groups (bacterial FDHs, AaFDH, MsFDH, PsFDH, and TsFDH, vs. eukaryotic FDHs, yeast CbFDH and fungal CsFDH) as previously described [40]. Bacterial FDHs had at least 82.5% sequence identity, and CbFDH and CsFDH shared 61.2% sequence identity. Although all the amino acids critical for catalysis or cofactor binding are highly conserved [51] in both FDH groups, bacterial FDHs contained an elongated N-terminal loop and C-terminal loop compared to eukaryotic FDHs (Fig. 5). The N-terminal loop may be involved in the determination of the kinetic mechanism, i.e., an ordered or a random model, and the C-terminal loop may contribute to the better CO_2_-reducing activities of bacterial FDHs than those of eukaryotic FDHs. To understand the amino acid differences of FDHs at molecular level, the structural alignment of TsFDH and CbFDH was performed. The structure of TsFDH was modeled using SWISS-MODEL homology modeling [52]. The holo-crystal structure of NAD-dependent FDH from *Pseudomonas* sp. 101 (PdFDH, pdb code: 2NAD) was used as a template (92.0% sequence identity with TsFDH) for homology modeling of TsFDH structure because the C-terminal loop, which covers the substrate channel, is only present in the holo-structure: the loop may be largely fluctuated in the apo-structure (pdb code: 2NAC). The N-terminal loop covers a significant part of the enzyme (Fig. 6A), and some amino acids in the loop interact with other amino acids of the subunit or the other chain in a dimeric form (structures not shown). However, the N-terminal loop does not have direct interactions with the substrate binding pocket. It was reported that PdFDH has a narrow substrate channel, and Arg284 on the wall of the substrate channel provides conformational mobility for binding and delivery of substrates [53]. In addition, Arg284 has close contacts with an inhibitor i.e. azide in the ternary complex structure (enzyme-NAD-azide, pdb code: 2NAD). Based on this structure information of PdFDH, it can be speculated that upon sequential binding of cofactor and substrate the C-terminal loop can be formed and then, contribute to conformational changes of the substrate channel including Arg284 for enzyme catalysis. The C-terminal loop, which is not present in the structure of CbFDH, in the modeled structure of TsFDH also covers the substrate binding channel, including Arg284 (Fig. 6B). This structural feature of TsFDH may be associated with CO_2_ accessibility or binding to the active site given that the kinetics data revealed that TsFDH exhibited a better *K*_B_ value than CbFDH (Table 1). However, we do not yet know whether the loops play important roles in CO_2_ binding or catalytic motion in the bacterial FDHs. Although there are many crystal structures and abundant biochemical information on NAD-dependent FDHs, the functions of these loops remains unclear. We plan to prepare a TsFDH C-terminal loop deletion mutant to test the hypothesis.

In summary, five FDHs with acidic optimum pH identified from biochemical data were tested for CO_2_ reduction. The superior CO_2_-reducing activity of TsFDH was confirmed by enzyme kinetics and formate production from CO_2_ gas. We propose that TsFDH is an alternative to the conventional CO_2_-reducing biocatalyst CbFDH. However, further experiments, including protein engineering and the development of NADH-regeneration systems, will be required to improve the CO_2_-reducing efficiency of TsFDH.

## Supporting Information

Figure S1 **Thermal unfolding curves of the FDHs measured by the DSF method.** AaFDH (blue), CbFDH (red), CsFDH (green), MsFDH(magenta), PsFDH (cyan), and TsFDH (dark gray).
(TIF)

Figure S2 **Michaelis-Menten plot for kinetic study.** Michaelis-Menten plots of TsFDH-catalyzed A) formate oxidation and B) CO_2_ reduction and CbFDH-catalyzed C) formate oxidation and D) CO_2_ reduction.
(TIF)

Figure S3 **Lineweaver-Burk plot for kinetic study.** Double reciprocal plots of initial rates of TsFDH-catalyzed A) formate oxidation and B) CO_2_ reduction; CbFDH-catalyzed C) formate oxidation and D) CO_2_ reduction with various sodium formate and sodium bicarbonate concentrations (•: 10 mM, ▴: 20 mM, ▾: 30 mM, ▪: 50 mM). a) and b) in insets show the secondary plots of the slopes and intercepts against the reciprocal concentration of invariant substrate, respectively.
(TIF)

Table S1 **The primers used in this study.**
(DOCX)

Table S2 **Comparison of the characteristics of TsFDH and other CO_2_ reductases.**
(DOCX)

Table S3 **Biochemical properties of the FDHs studied in this study.**
(DOCX)

## References

[1] Baskaya FS, Zhao X, Flickinger MC, Wang P (2010) Thermodynamic feasibility of enzymatic reduction of carbon dioxide to methanol. Appl Biochem Biotechnol 162: 391–398. 19763899 10.1007/s12010-009-8758-x

[2] Crable BR, Plugge CM, McInerney MJ, Stams AJM (2011) Formate formation and formate conversion in biological fuels production. Enzyme Res 2011: 532536 doi: 10.4061/2011/532536 21687599 10.4061/2011/532536 PMC3112519

[3] Tong X, El-Zahab B, Zhao X, Liu Y, Wang P (2011) Enzymatic synthesis of L-lactic acid from carbon dioxide and ethanol with an inherent cofactor regeneration cycle. Biotechnol Bioeng 108: 465–469. 20830681 10.1002/bit.22938

[4] El-Zahab B, Donnelly D, Wang P (2008) Particle-tethered NADH for production of methanol from CO_2_ catalyzed by coimmobilized enzymes. Biotechnol Bioeng 99: 508–514. 17680680 10.1002/bit.21584

[5] Lee HJ, Lee SH, Park CB, Won K (2011) Coenzyme analogs: Excellent substitutes (not poor imitations) for electrochemical regeneration. Chem Commun 47: 12538–12540. 10.1039/c1cc14313a 22003495

[6] Jung WS, Han J, Ha S (2007) Analysis of palladium-based anode electrode using electrochemical impedance spectra in direct formic acid fuel cells. J Power Sources 173: 53–59.

[7] Sakakura T, Choi JC, Yasuda H (2007) Transformation of carbon dioxide. Chem Rev 107: 2365–2387. 17564481 10.1021/cr068357u

[8] North M, Pasquale R, Young C (2010) Synthesis of cyclic carbonates from epoxides and CO_2_ . Green Chem 12: 1514–1539.

[9] Federsel C, Ziebart C, Jackstell R, Baumann W, Beller M (2012) Catalytic hydrogenation of carbon dioxide and bicarbonates with a well-defined cobalt dihydrogen complex. Chem Eur J 18: 72–75. 22147509 10.1002/chem.201101343

[10] Park S, Bézier D, Brookhart M (2012) An efficient iridium catalyst for reduction of carbon dioxide to methane with trialkylsilanes. J Am Chem Soc 134: 11404–11407. 22765847 10.1021/ja305318c

[11] Sekizawa K, Maeda K, Domen K, Koike K, Ishitani O (2013) Artificial Z-scheme constructed with a supramolecular metal complex and semiconductor for the photocatalytic reduction of CO_2_ . J Am Chem Soc 135: 4596–4599. 23470246 10.1021/ja311541a PMC3679556

[12] Kanth BK, Lee J, Pack SP (2013) Carbonic anhydrase: Its biocatalytic mechanisms and functional properties for efficient CO_2_ capture process development. Eng Life Sci 13: 422–431.

[13] Jo BH, Kim IG, Seo JH, Kang DG, Cha HJ (2013) Engineered *Escherichia coli* with periplasmic carbonic anhydrase as a biocatalyst for CO_2_ sequestration. Appl Environ Microbiol 79: 6697–6705. 23974145 10.1128/AEM.02400-13 PMC3811487

[14] Kim S, Lee GY, Lee J, Rajkumar E, Baeg JO, et al (2013) Efficient electrochemical regeneration of nicotinamide cofactors using a cyclopentadienyl-rhodium complex on functionalized indium tin oxide electrodes. Electrochim Acta 96: 141–146.

[15] Parkinson BA, Weaver PF (1984) Photoelectrochemical pumping of enzymatic CO_2_ reduction. Nature 309: 148–149.

[16] Yadav RK, Baeg JO, Oh GH, Park NJ, Kong KJ, et al (2012) A photocatalyst-enzyme coupled artificial photosynthesis system for solar energy in production of formic acid from CO_2_ . J Am Chem Soc 134: 11455–11461. 22769600 10.1021/ja3009902

[17] Almendra MJ, Brondino CD, Gavel O, Pereira AS, Tavares P, et al (1999) Purification and characterization of a tungsten-containing formate dehydrogenase from *Desulfovibrio gigas* . Biochemistry 38: 16366–16372. 10587462 10.1021/bi990069n

[18] Cone JE, Martin Del Rio R, Davis JN, Stadtman TC (1976) Chemical characterization of the selenoprotein component of clostridial glycine reductase: Identification of selenocysteine as the organoselenium moiety. Proc Natl Acad Sci U S A 73: 2659–2663. 1066676 10.1073/pnas.73.8.2659 PMC430707

[19] Graentzdoerffer A, Rauh D, Pich A, Andreesen JR (2003) Molecular and biochemical characterization of two tungsten- and selenium-containing formate dehydrogenases from *Eubacterium acidaminophilum* that are associated with components of an iron-only hydrogenase. Arch Microbiol 179: 116–130. 12560990 10.1007/s00203-002-0508-1

[20] Reda T, Plugge CM, Abram NJ, Hirst J (2008) Reversible interconversion of carbon dioxide and formate by an electroactive enzyme. Proc Natl Acad Sci U S A 105: 10654–10658. 18667702 10.1073/pnas.0801290105 PMC2491486

[21] Bommarius AS, Schwarm M, Drauz K (1998) Biocatalysis to amino acid-based chiral pharmaceuticals - Examples and perspectives. J Mol Catal B: Enzym 5: 1–11.

[22] Gröger H, Hummel W, Rollmann C, Chamouleau F, Hüsken H, et al (2004) Preparative asymmetric reduction of ketones in a biphasic medium with an (S)-alcohol dehydrogenase under *in situ*-cofactor-recycling with a formate dehydrogenase. Tetrahedron 60: 633–640.

[23] Van Der Donk WA, Zhao H (2003) Recent developments in pyridine nucleotide regeneration. Curr Opin Biotechnol 14: 421–426. 12943852 10.1016/s0958-1669(03)00094-6

[24] Weckbecker A, Hummel W (2004) Improved synthesis of chiral alcohols with *Escherichia coli* cells co-expressing pyridine nucleotide transhydrogenase, NADP^+^-dependent alcohol dehydrogenase and NAD^+^-dependent formate dehydrogenase. Biotechnol Lett 26: 1739–1744. 15604828 10.1007/s10529-004-3746-2

[25] Kim T, Joo JC, Yoo YJ (2012) Hydrophobic interaction network analysis for thermostabilization of a mesophilic xylanase. J Biotechnol 161: 49–59. 22642881 10.1016/j.jbiotec.2012.04.015

[26] Phillips K, de la Peña AH (2011) The combined use of the Thermofluor assay and ThermoQ analytical software for the determination of protein stability and buffer optimization as an aid in protein crystallization. Curr Protoc Mol Biol Chapter 10 : Unit 10.28. 10.1002/0471142727.mb1028s94 21472694

[27] Peacock D, Boulter D (1970) Kinetic studies of formate dehydrogenase. Biochem J 120: 763–769. 4322039 10.1042/bj1200763 PMC1179669

[28] Cleland WW (1963) The kinetics of enzyme-catalyzed reactions with two or more substrates or products. I. Nomenclature and rate equations. Biochim Biophys Acta 67: 104–137. 14021667 10.1016/0006-3002(63)91800-6

[29] Nanba H, Takaoka Y, Hasegawa J (2003) Purification and characterization of formate dehydrogenase from *Ancylobacter aquaticus* strain KNK607M, and cloning of the gene. Biosci Biotechnol Biochem 64: 720–728. 10.1271/bbb.67.720 12784610

[30] Watanabe T, Fujiwara T, Umezawa T, Shimada M, Hattori T (2008) Cloning of a cDNA encoding a NAD-dependent formate dehydrogenase involved in oxalic acid metabolism from the white-rot fungus *Ceriporiopsis subvermispora* and its gene expression analysis. FEMS Microbiol Lett 279: 64–70. 18177307 10.1111/j.1574-6968.2007.01022.x

[31] Shinoda T, Satoh T, Mineki S, Iida M, Taguchi H (2002) Cloning, nucleotide sequencing, and expression in *Escherichia coli* of the gene for formate dehydrogenase of *Paracoccus* sp. 12-A, a formate-assimilating bacterium. Biosci Biotechnol Biochem 66: 271–276. 11999398 10.1271/bbb.66.271

[32] Nanba H, Takaoka Y, Hasegawa J (2003) Purification and characterization of an α-haloketone-resistant formate dehydrogenase from *Thiobacillus* sp. strain KNK65MA, and cloning of the gene. biosci Biotechnol Biochem 67: 2145–2153. 14586102 10.1271/bbb.67.2145

[33] Chenault HK, Whitesides GM (1987) Regeneration of nicotinamide cofactors for use in organic synthesis. Appl Biochem Biotechnol 14: 147–197. 3304160 10.1007/BF02798431

[34] De Bok FAM, Hagedoorn PL, Silva PJ, Hagen WR, Schiltz E, et al (2003) Two W-containing formate dehydrogenases (CO_2_-reductases) involved in syntrophic propionate oxidation by *Syntrophobacter fumaroxidans* . Eur J Biochem 270: 2476–2485. 12755703 10.1046/j.1432-1033.2003.03619.x

[35] Axley MJ, Bock A, Stadtman TC (1991) Catalytic properties of an *Escherichia coli* formate dehydrogenase mutant in which sulfur replaces selenium. Proc Natl Acad Sci U S A 88: 8450–8454. 1924303 10.1073/pnas.88.19.8450 PMC52526

[36] Chen GT, Axley MJ, Hacia J, Inouye M (1992) Overproduction of a selenocysteine-containing polypeptide in *Escherichia coli*: The *fdhF* gene product. Mol Microbiol 6: 781–785. 1533438 10.1111/j.1365-2958.1992.tb01528.x

[37] Gladyshev VN, Boyington JC, Khangulov SV, Grahame DA, Stadtman TC, et al (1996) Characterization of crystalline formate dehydrogenase H from *Escherichia coli*: Stabilization, EPR spectroscopy, and preliminary crystallographic analysis. J Biol Chem 271: 8095–8100. 8626495 10.1074/jbc.271.14.8095

[38] Schuchmann K, Muller V (2013) Direct and reversible hydrogenation of CO_2_ to formate by a bacterial carbon dioxide reductase. Science 342: 1382–1385. 24337298 10.1126/science.1244758

[39] Scheer M, Grote A, Chang A, Schomburg I, Munaretto C, et al (2011) BRENDA, the enzyme information system in 2011. Nucleic Acids Res 39: D670–D676. 21062828 10.1093/nar/gkq1089 PMC3013686

[40] Tishkov VI, Popov VO (2004) Catalytic mechanism and application of formate dehydrogenase. Biochemistry (Moscow) 69: 1252–1267. 15627379 10.1007/s10541-005-0071-x

[41] Ordu EB, Sessions RB, Clarke AR, Karagüler NG (2013) Effect of surface electrostatic interactions on the stability and folding of formate dehydrogenase from *Candida methylica* . J Mol Catal B: Enzym 95: 23–28.

[42] Hayashi H, Ogo S, Abura T, Fukuzumi S (2003) Accelerating effect of a proton on the reduction of CO_2_ dissolved in water under acidic conditions. Isolation, crystal structure, and reducing ability of a water-soluble ruthenium hydride complex. J Am Chem Soc 125: 14266–14267. 14624561 10.1021/ja036117f

[43] Huang YH, Zhang TC (2004) Effects of low pH on nitrate reduction by iron powder. Water Res 38: 2631–2642. 15207593 10.1016/j.watres.2004.03.015

[44] Kumar B, Llorente M, Froehlich J, Dang T, Sathrum A, et al (2012) Photochemical and photoelectrochemical reduction of CO_2_ . Annu Rev Phys Chem 63: 541–569. 22404587 10.1146/annurev-physchem-032511-143759

[45] Kim S, Kim MK, Lee SH, Yoon S, Jung KD (2014) Conversion of CO_2_ to formate in an electroenzymatic cell using *Candida boidinii* formate dehydrogenase. J Mol Catal B: Enzym 102: 9–15.

[46] Wichmann R, Vasic-Racki D (2005) Cofactor regeneration at the lab scale. Adv Biochem Eng Biotechnol 92: 225–260. 15791939 10.1007/b98911

[47] Wu JT, Wu LH, Knight JA (1986) Stability of NADPH: Effect of various factors on the kinetics of degradation. Clin Chem 32: 314–319. 3943190

[48] Lee HJ, Lee SH, Park CB, Won K (2011) Coenzyme analogs: excellent substitutes (not poor imitations) for electrochemical regeneration. Chem Commun 47: 12538–12540. 10.1039/c1cc14313a 22003495

[49] Larkin MA, Blackshields G, Brown NP, Chenna R, Mcgettigan PA, et al (2007) Clustal W and Clustal X version 2.0. Bioinformatics 23: 2947–2948. 17846036 10.1093/bioinformatics/btm404

[50] Gouet P, Courcelle E, Stuart DI, Métoz F (1999) ESPript: Analysis of multiple sequence alignments in PostScript. Bioinformatics 15: 305–308. 10320398 10.1093/bioinformatics/15.4.305

[51] Popov VO, Lamzin VS (1994) NAD^+^-dependent formate dehydrogenase. Biochem J 301: 625–643. 8053888 10.1042/bj3010625 PMC1137035

[52] Schwede T, Kopp J, Guex N, Peitsch MC (2003) SWISS-MODEL: An automated protein homology-modeling server. Nucleic Acids Res 31: 3381–3385. 12824332 10.1093/nar/gkg520 PMC168927

[53] Nilov DK, Shabalin IG, Popov VO, Švedas VK (2011) Investigation of formate transport through the substrate channel of formate dehydrogenase by steered molecular dynamics simulations. Biochemistry (Moscow) 76: 172–174. 21568849 10.1134/s0006297911020027

