# Supplementary figures and images for: Efficient CO2-Reducing Activity of NAD-Dependent Formate Dehydrogenase from Thiobacillus sp. KNK65MA for Formate Production from CO2 Gas
Source: PLoS One. 2014 Jul 25;9(7):e103111. doi: 10.1371/journal.pone.0103111 (PMC4111417; doi:10.1371/journal.pone.0103111)

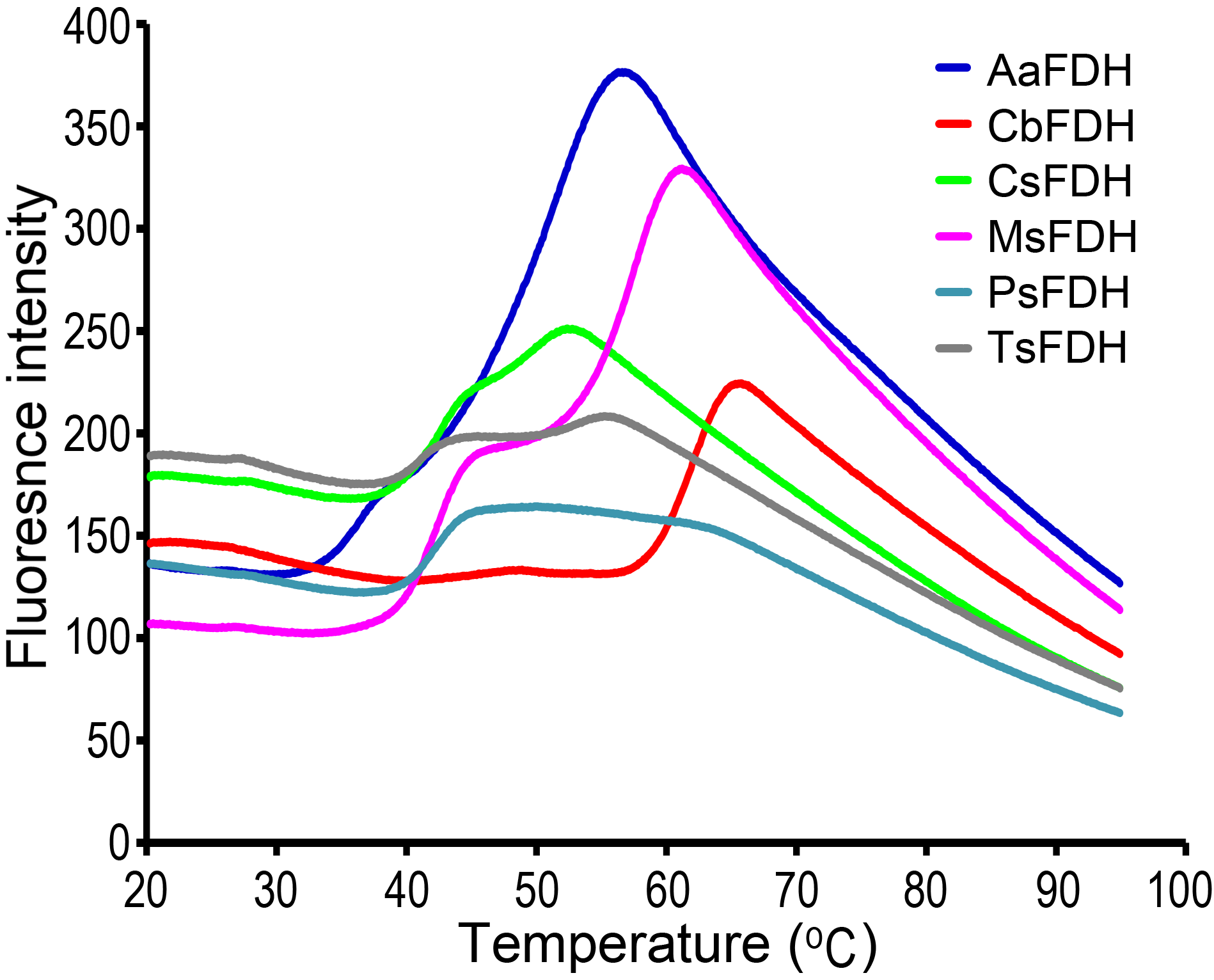

Supplement: Figure S1 — Thermal unfolding curves of the FDHs measured by the DSF method. AaFDH (blue), CbFDH (red), CsFDH (green), MsFDH(magenta), PsFDH (cyan), and TsFDH (dark gray). (TIF) [file pone.0103111.s001.tif]

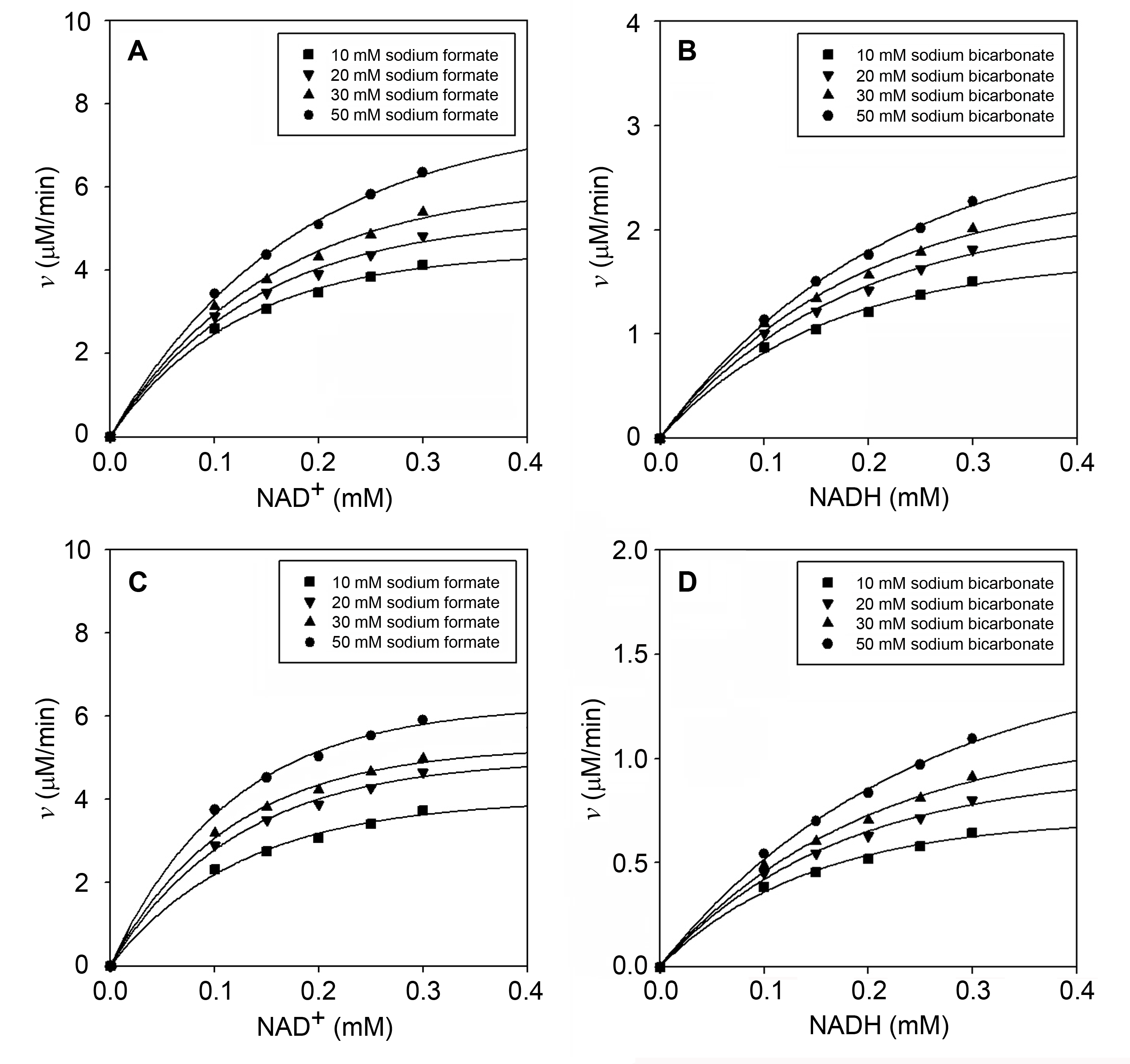

Supplement: Figure S2 — Michaelis-Menten plot for kinetic study. Michaelis-Menten plots of TsFDH-catalyzed A) formate oxidation and B) CO2 reduction and CbFDH-catalyzed C) formate oxidation and D) CO2 reduction. (TIF) [file pone.0103111.s002.tif]

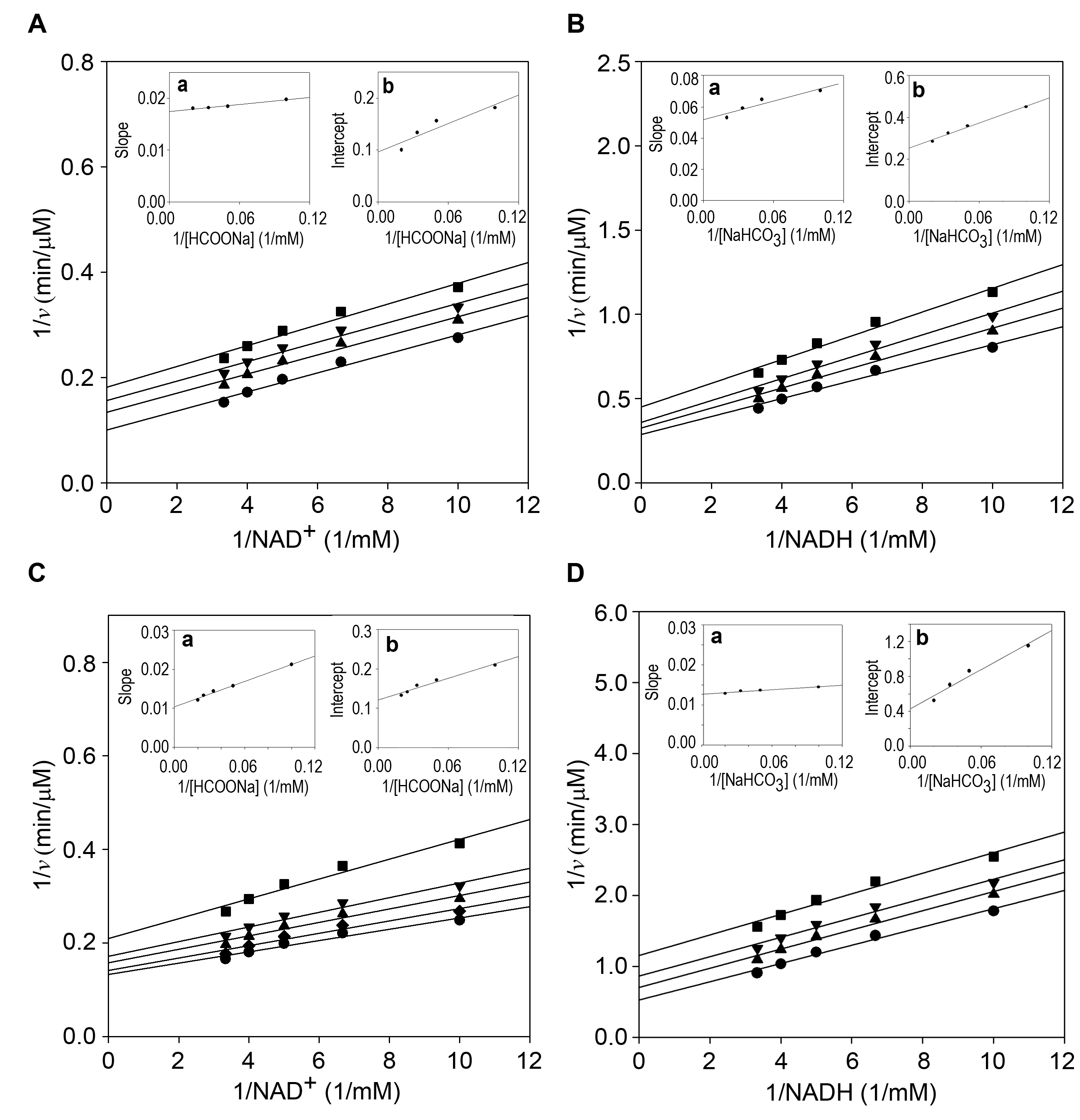

Supplement: Figure S3 — Lineweaver-Burk plot for kinetic study. Double reciprocal plots of initial rates of TsFDH-catalyzed A) formate oxidation and B) CO2 reduction; CbFDH-catalyzed C) formate oxidation and D) CO2 reduction with various sodium formate and sodium bicarbonate concentrations (•: 10 mM, ▴: 20 mM, ▾: 30 mM, ▪: 50 mM). a) and b) in insets show the secondary plots of the slopes and intercepts against the reciprocal concentration of invariant substrate, respectively. (TIF) [file pone.0103111.s003.tif]
