# Supplementary material for: Efficient CO2-Reducing Activity of NAD-Dependent Formate Dehydrogenase from Thiobacillus sp. KNK65MA for Formate Production from CO2 Gas
Source: PLoS One. 2014 Jul 25;9(7):e103111. doi: 10.1371/journal.pone.0103111 (PMC4111417; doi:10.1371/journal.pone.0103111)
Supplement: Table S1 — The primers used in this study. (DOCX) [file pone.0103111.s004.docx]

**Table S1. The primers used in this study.**

| FDHs | Primers |
| --- | --- |
| AaFDH | Forward: GGG CCC GCT AGC ATG GCG AAG GTT CTG TGC |
|  | Reverse: CCC GGG GAA TTC TTA ATG ATG GTG GTG ATG |
| CbFDH | Forward: GGG CCC GCT AGC ATG AAG ATC GTT TTA GTC |
|  | Reverse: CCC GGG GAA TTC TTA ATG ATG GTG GTG ATG |
| CsFDH | Forward: GGG CCC GCT AGC ATG AAG GTT CTT GCT ATT |
|  | Reverse: CCC GGG GAA TTC TTA ATG ATG GTG GTG ATG |
| MsFDH | Forward: GGG CCC GCT AGC ATG GCC AAG GTT GTT TGC |
|  | Reverse: CCC GGG GAA TTC TTA ATG ATG GTG GTG ATG |
| PsFDH | Forward: GGG CCC GCT AGC ATG GCC AAG GTA GTT TGC |
|  | Reverse: CCC GGG GAA TTC TTA ATG ATG GTG GTG ATG |
| TsFDH | Forward: GGG CCC GCT AGC ATG GCG AAA ATA CTT TGC |
|  | Reverse: CCC GGG GAA TTC TTA ATG ATG GTG GTG ATG |
