# Supplementary material for: Efficient CO2-Reducing Activity of NAD-Dependent Formate Dehydrogenase from Thiobacillus sp. KNK65MA for Formate Production from CO2 Gas
Source: PLoS One. 2014 Jul 25;9(7):e103111. doi: 10.1371/journal.pone.0103111 (PMC4111417; doi:10.1371/journal.pone.0103111)
Supplement: Table S2 — Comparison of the characteristics of TsFDH and other CO2 reductases. (DOCX) [file pone.0103111.s005.docx]

**Table S2. Comparison of the characteristics of TsFDH and other CO2 reductases.**

| Enzyme | Organism | Electron donors | Metal & cofactors | O_2_ sensitivity | Recombinant expression | Turnover rate (1/s) | Ref. |
| --- | --- | --- | --- | --- | --- | --- | --- |
| FDH | *Thiobacillus* sp. Strain KNK65MA | NADH | - | Insensitive | *E. coli* | 0.32 | This work |
| FDH1 | *Syntrophobacter fumaroxidans* | MV | W, Fe-S clusters, Selenocysteine | Very sensitive | - | 282 | 20 |
| HDCR | *Acetobacterium woodii* | H_2_, MV | Mo, Zn, Fe-S clusters, Selenocysteine | Very sensitive | - | 28 | 38 |

MV: Methyl viologen.

HDCR: Hydrogen-dependent carbon dioxide reductase.
