# Supplementary material for: Efficient CO2-Reducing Activity of NAD-Dependent Formate Dehydrogenase from Thiobacillus sp. KNK65MA for Formate Production from CO2 Gas
Source: PLoS One. 2014 Jul 25;9(7):e103111. doi: 10.1371/journal.pone.0103111 (PMC4111417; doi:10.1371/journal.pone.0103111)
Supplement: Table S3 — Biochemical properties of the FDHs studied in this study. (DOCX) [file pone.0103111.s006.docx]

**Table S3. Biochemical properties of the FDHs studied in this study.**

| FDHs | Optimum pH^[a]^ | | Activity at optimum pH^[a]^ | | T_m_ values^[c]^  [°C] |
| --- | --- | --- | --- | --- | --- |
|  | Formate oxidation | CO_2_ reduction^[b]^ | Formate oxidation [U/mg enzyme] | CO_2_ reduction^[b]^ [mU/mg enzyme] |  |
| AaFDH | 6.0 | 6.0 | 21.6±0.23 | 4.5±0.22 | 51.2±0.34 |
| CbFDH | 7.0 | 5.5 | 6.1±0.02 | 1.6±0.08 | 61.8±0.34 |
| CsFDH | 6.5 | 6.0 | 1.3±0.10 | 0.8±0.03 | 49.9±0.34 |
| MsFDH | 5.5 | 5.5 | 14.3±0.10 | 2.8±0.12 | 57.5±0.34 |
| PsFDH | 5.5 | 5.5 | 12.2±0.40 | 6.5±0.06 | 42.3±0.34 |
| TsFDH | 6.5 | 5.5 | 10.9±0.34 | 12.2±0.13 | 53.2±0.34 |

[a] These values were determined from the results in Fig. 2.

[b] The activity of CO_2_ reduction was determined using sodium bicarbonate as described in Materials and methods.

[c] The T_m_ values were calculated from the results in Fig. S1.
